# Mechanical Ventilation of Hospital Wards

**Published:** 1896-07-18

**Authors:** 


### MECHANICAL VENTILATION OF HOSPITAL WARDS.

The editor of the *Builder* writes: In an article under the above heading in your issue of July 11th, you say that "a writer in the *Builder*, who signs himself F.R.C.S., urges," etc. The phrase conveys a wrong impression. "F.R.C.S." is merely the writer of a letter to the *Builder*, which is quite a different thing. His communication was not accepted as an article; it was merely inserted in the correspondence column.

